# Dermatological manifestations in autoimmune thyroid disorders

**DOI:** 10.1186/1753-6561-6-S4-O39

**Published:** 2012-07-09

**Authors:** N Nagaraj, AL Balaji, S Singla, M Prakash

**Affiliations:** 1 Rajiv Gandhi University of Health Sciences, India

## Introduction

Thyroid hormone deficiency causes skin changes including thickening, pruritis, alopecia, xerosis, nail changes and dermopathy. These manifestations depend on various factors like duration of disease, treatment and follow-up. We conducted this study to show the cutaneous manifestations of auto-immune thyroid disease (AITD) as a marker for diagnosis and treatment.

## Methods

We examined 400 consecutive patients presenting to the endocrinology/dermatology department of a tertiary care referral hospital in Bangalore who were diagnosed with AITD between the periods from 06/07 – 06/10 on an outpatient basis. Detailed history and physical examination including cutaneous examination was done. Mean follow up was done for the period of 6 months for the cutaneous manifestations.

## Results

400/1020 (39.2%) patients presented with cutaneous manifestations with AITD. This comprised of 42(10.5%) males and 358(89.5%) females. The cutaneous presentations were nail changes(336), dermopathy(48), urticaria(114), vitiligo(74), alopecia(90), and pruritis(242). The other co-morbid conditions associated were diabetes(160), hypertension(190), psoriasis(38), addisons disease(9) and PCOS (93).

## Conclusions

The study shows the cutaneous manifestations of AITD, which should be evaluated completely and followed-up with regular thyroid function tests and thyroid hormone replacement. Discontinuation of thyroid hormone therapy may result in recurrence. The cutaneous manifestations can also be used as a diagnostic marker for AITD. In a country like India, thyroid diseases go unnoticed mainly due to reduced incidence previously and lack of awareness among the patient population. AITD is not easily diagnosed and cutaneous manifestations shall result in early diagnosis and treatment for AITD and other thyroid diseases.

